# Methods of prolonging the effect of caudal block in children

**DOI:** 10.3389/fped.2024.1406263

**Published:** 2024-06-03

**Authors:** Weiyi Xu, Haixu Wei, Tao Zhang

**Affiliations:** Department of Anesthesiology, The First Affiliated Hospital, Sun Yat-sen University, Guangzhou, Guangdong, China

**Keywords:** anesthesia, caudal, epidural, analgesia, child

## Abstract

Caudal epidural blockade is one of the most frequently administered regional anesthesia techniques in children. It is a supplement during general anesthesia and for providing postoperative analgesia in pediatrics for sub-umbilical surgeries, especially for genitourinary surgeries. However, the duration of the analgesic effect is occasionally unsatisfactory. In this review, we discuss the main advantages and disadvantages of different techniques to prolong postoperative analgesia for single-injection caudal blockade in children. A literature search of the keywords “caudal”, “analgesia”, “pediatric”, and “children” was performed using PubMed and Web of Science databases. We highlight that analgesic quality correlates substantially with the local anesthetic's type, dose, the timing relationship between caudal block and surgery, caudal catheterization, and administration of epidural opioids or other adjuvant drugs.

## 1 Introduction

Caudal epidural analgesia is one of the most well-liked and frequently used regional blocks in pediatric anesthesia. It is a dependable and secure approach that offers patients undergoing genitourinary, abdominal, and lower limb surgery good intra- and postoperative analgesia, making it a valuable complement to general anesthesia. According to data from more than 100,000 neuraxial and peripheral nerve blocks performed from 2007 to 2015 at more than 20 children's hospitals on the incidence of major complications of pediatric regional anesthesia, the most common procedure was caudal block across all age groups under 18 years old, and no permanent neurologic deficits were reported (1). It also demonstrates no difference between peripheral and neuraxial blocks regarding the risk of neurologic complications. General anesthesia combined with caudal block could reduce opioid consumption, enable earlier tracheal extubation, and facilitate the resumption of spontaneous ventilation (2, 3).

Although the caudal block is versatile, one significant drawback of the single injection is the relatively short duration of analgesia. The topic of extending the duration of single-injection caudal analgesia has long been researched. This review summarizes the most recent methods of prolonging the period of single-injection caudal analgesia and weighs the possible advantages of each approach against the perceived concerns it carries in children.

**Figure 1:**
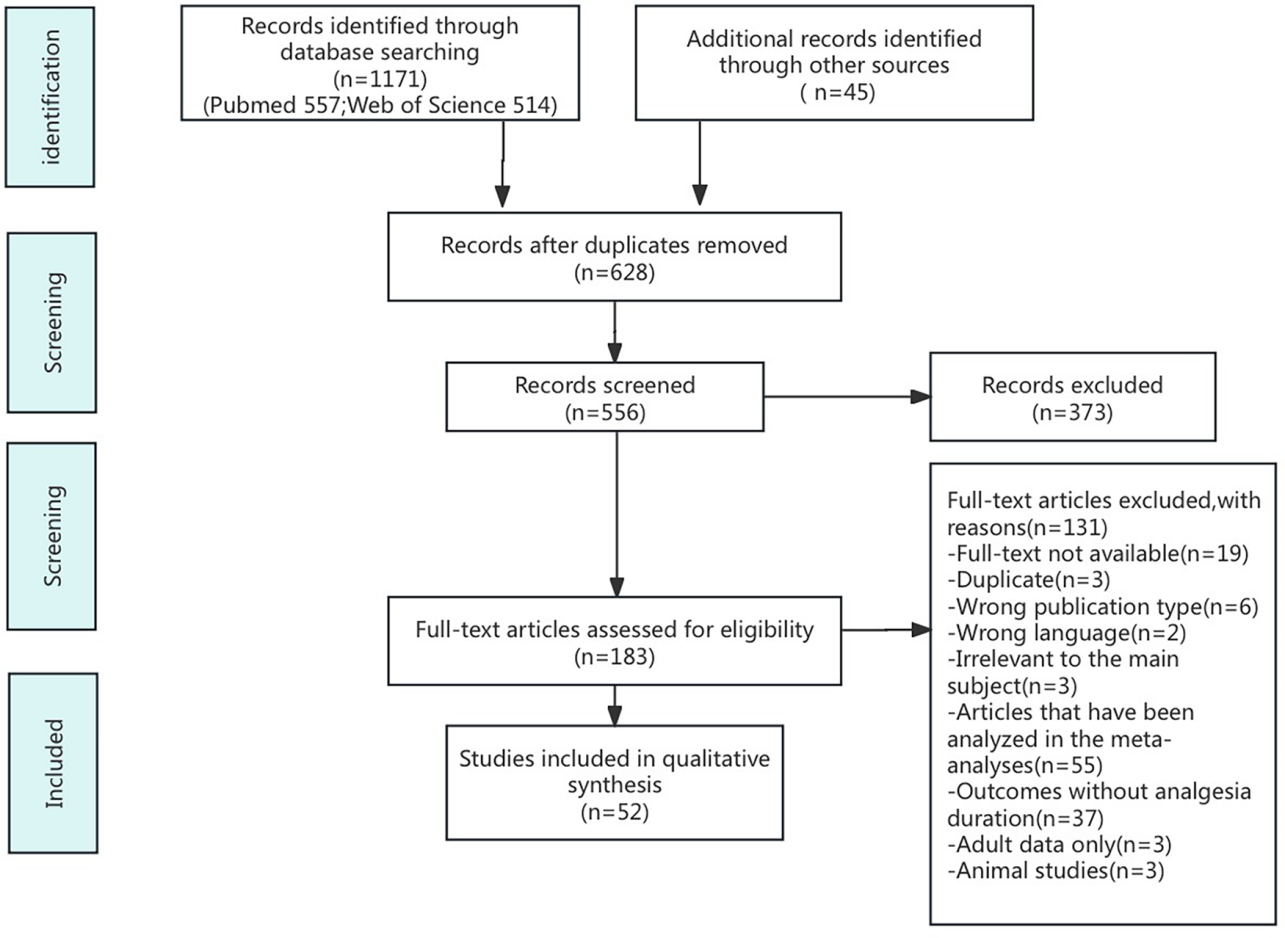
PRISMA flow chart of search and selection of papers.

A literature search was performed using PubMed and Web of Science databases to review the methods of prolonging the effect of single caudal block in children. The search words used were “caudal”, “analgesia”, “pediatric”, and “children”. Boolean operators were used to combine the search words as follows: caudal AND analgesia AND (children OR pediatric). The search limits were English language, publication date from 1995 till present, and article type, including systematic reviews, meta-analyses, randomized controlled trials, and observational studies. Abstracts were screened, duplicates were removed, and relevant articles were selected. We included articles that were specific to the postoperative analgesic quality of single caudal block and those that reported outcomes in terms of analgesia time. We excluded animal studies and articles that were analyzed in the meta-analyses (Figure 1).

## 2 Local anesthetics

### 2.1 Bupivacaine vs. Ropivacaine

Bupivacaine and ropivacaine are efficient and long-acting amide local anesthetics. They are frequently used for caudal epidural analgesia in children because of their prolonged duration of action and ability to provide reliable anesthesia and analgesia (4). Bupivacaine is often a racemic combination of R- and S-enantiomers because of chiral carbon atoms; ropivacaine is the first local anesthetic to be synthesized as a pure S-enantiomer. Because the S-enantiomer has a lower affinity for sodium and potassium (hKvl.5) cardiac channels than the R(+)-enantiomer does for these cardiac channels (5), ropivacaine has a wider margin of safety than bupivacaine (6, 7). It is more slowly absorbed systematically from the caudal epidural space, and has a lower potential for adverse effects on the central nervous system and the cardiovascular system (4, 8).

Although administration of amide local anesthetics into the caudal epidural space has been our standard method of providing postoperative analgesia for surgeries below the umbilicus, there is the risk for local anesthetic systemic toxicity (LAST), especially in neonates and infants due to their immature cytochrome P450 (CYP) system and lower plasma levels of a1-acid glycoprotein (9, 10). The CYP system will not develop to adult metabolic capability until adolescence (9). Bupivacaine is metabolized by the CYP3A4 subtype and reaches maximum clearance at 12 months of age, while ropivacaine is metabolized by CYP1A2, which is not fully mature before the age of 3 years. This may result in decreased drug clearance, an extended terminal half-life, and the potential for local anesthetic plasma accumulation. The a1-acid glycoprotein has the capacity to bind amide local anesthetic agents in plasma, and the concentration is significantly low at birth, contributing to an increased free fraction of the drug in newborns, thereby potentiating the risk of toxicity (4). However, esters such as 2-chloroprocaine undergo metabolism rapidly in the plasma by serum cholinesterases. This results in a significantly shorter serum half-life compared with the amides and, therefore, minimizes the accumulation of local anesthetics in plasma and reduces the risk of toxicity in patients of all ages, including newborns and infants. Lowering the dosage of local anesthetics for children under two is another recommendation made by the American Society of Regional Anesthesia and Pain Medicine and the European Society of Regional Anaesthesia and Pain Therapy (4). Their higher cardiac output tends to speed up the systemic absorption of local anesthetics, resulting in higher initial plasma concentrations, shorter durations of action, and more susceptibility to the LAST.

Overall, these findings suggest that caudal anesthesia with ropivacaine in pediatric patients is safe and effective for pediatric patients, with fewer side effects in the postoperative period. However, because of the immaturity of the metabolism, continuous application of long-acting amide local anesthetics in newborns and infants requires caution.

### 2.2 Volume

The quality and level of the caudal block are correlated substantially with the volume and concentration of the local anesthetic solution. Surprisingly, the analgesic duration depends more on the solution distribution level than the concentration of local anesthetic after caudal block (11). The analgesic duration may be prolonged because a larger volume of local anesthetics will provide a higher initial level of blockage. In a prospective, randomized, observer-blind study comparing the analgesia quality between the high volume/low concentration (HVLC:0.15% 1.5 ml/kg) and low volume/high concentration ropivacaine (LVHC:0.225% 1 ml/kg) for unilateral orchiopexy surgeries (12), the results show the median with IQR of local anesthetics spread level of ropivacaine confirmed by fluoroscopic examination for LVHC group (*n* = 37) was T11 (T8–L2), and was T6 (T3–T11) for the HVLC group (*n* = 36). The cases in the HVLC group received significantly longer analgesic duration (554.5 min vs. 363.0 min) and fewer rescue analgesia requirements in the first 24 h after surgery (50.0% vs. 75.7%). It also showed the result of the parameter estimation of linear regression model between postoperative oral acetaminophen time(Y) and spread level of ropivacaine (X), the analgesic duration may prolong with the block level respectively in each group (HVLC: *R*^2^ = 0.429, *P* < 0.01; LVHC: *R*^2^ = 0.203, *P* < 0.05).

The lumbar level had a substantially larger amount of epidural space per segment than the thoracic and caudal levels. The median volumes of the epidural space per vertebral segment were 0.60 ml (95%CI 0.38–0.75) for thoracic,1.18 ml (95%CI 0.94–1.43) for lumbar, and 0.85 ml (95%CI 0.56–1.18) for caudal (13). The current recommendations for the well-established weight-based doses for caudally injected 0.2% bupivacaine were introduced by Armitage—whereby 0.5 ml/kg may reach sacral, 1.0 ml/kg to lumbar, and 1.25 ml/kg to mid-thoracic dermatomes (14). The efficacy of this well-established formula of the caudal epidural block has also been explored on ropivacaine. L. Brenner and colleagues (15) reported that the ultrasound-assessed cranial spread (median, IQR) of receiving a caudal block with 0.7,1.0,1.3 ml/kg ropivacaine are L3(L2-L5), L2(L1-L3), L2(L1-L2)respectively. A significant difference was found between Groups 1.3 and 0.7 (*P* = 0.0002) and Group 1.0 (*P* = 0.03). However, no difference in cranial spread could be observed between Groups 0.7 and 1.0 (*P* = ns). A *post hoc* evaluation of the spread of the local anesthetics relative to weight, height, and BMI found weak inverse correlations about these factors in all study groups. It is also challenging to reasonably estimate the cranial spread of local anesthetic based on the volume of liquid injected because the cranial extension of even high-volume blocks (1.3 ml/kg) rarely reached above the thoracolumbar level, with a maximally observed level of T10. Additionally, clinical experience has shown that even high-volume caudal blocks (1.3–1.5 ml/kg) do not allow procedures such as umbilical hernia repair to be performed as an awake regional technique. This counters to the above quoted early studies may be because of the differences in cutaneous testing and visualizing by radiographic methods.

Caudal block with a high volume of local anesthetic may cause a greater intracranial pressure increase. Le and his colleagues (16) demonstrated that the administration of 1.5 ml/kg of local anesthetic for caudal block resulted in a more significant increase in intracranial pressure than the administration of 1.0 ml/kg of local anesthetic by measuring optic nerve sheath diameter which correlates with the degree of intracranial pressure. The highest value of optic nerve sheath diameter was observed 10 min after the caudal block. Thus, careful consideration should be given to children with intracranial pathologies, even with conventional volumes of local anesthetic for caudal block.

### 2.3 Concentration

Although the analgesic duration is more dependent on the level of solution distribution than on the local anesthetic concentration, analgesia has also been shown to be significantly prolonged when using more concentrated ropivacaine. However, a greater degree and longer duration of motor block also resulted. A double-blind, randomized, comparative study (17) indicated that the median time to first analgesic treatment in children scheduled for inguinal surgery was 3.3 h, 4.5 h, and 4.2 h after a single caudal block by 0.1%, 0.2% and 0.3% ropivacaine 1 ml/kg respectively. The incidence of motor block was also aggravated by the ropivacaine concentration used [5/38 (13%) compared with 10/36 (28%)]. Taking account of the balance between higher postoperative pain scores and complaints of leg weakness, the European Society of Regional Anaesthesia and Pain Therapy/American Society of Regional Anesthesia recommends ropivacaine 0.2% or levobupivacaine/bupivacaine 0.25% for the performance of caudal blocks in children and should not receive more than 2 mg/kg ropivacaine or 2.5 mg/kg bupivacaine or levobupivacaine (4).

Deng found that children of school age need a greater concentration of ropivacaine (0.143% vs. 0.107%) than children of preschool age to provide intra-operative caudal analgesia for elective hypospadias repair when combined with general anesthesia (18). Further studies for postoperative analgesia should be explored.

In the meantime, anesthesiologists must bear in mind the local anesthetic-induced systemic toxicity (LAST). Since more subtle toxic effects cannot be detected when under general anesthesia or deep sedation, LAST may appear as central nervous system indicators (seizures) or cardiovascular signals (ventricular arrhythmias or cardiac arrest).

## 3 Timepoint of caudal block

The time point of the caudal block performed is associated with the analgesic effect as well. Pre-emptive epidural analgesia could intercept nociceptive input, increase the threshold for nociception, and block or decrease nociceptor receptor activation (19, 20). Thus it produces superior analgesia effects with fewer analgesic demands and adverse effects, attenuates the surgery-induced immune alterations, and improves the postoperative recovery. Yang and colleagues (20) found that the time to prescribe pain rescue medications was later, protein and mRNA expressions of tumor necrosis factor-α, interleukin (IL)-6, and IL-8 were decreased, and that of IL-4 was increased in the pre-emptive group compared with the routine one in patients undergoing thoracotomy. However, pre-emptive analgesia remains debatable. A prospective, randomized, double-blind study concluded that preoperative epidural-infused local anesthetics and additives [sufentanyl, clonidine, and S(+)-ketamine] did not provide excellent pre-emptive analgesia (21).

Recently, the benefits of employing a double-caudal approach, in which the caudal is “topped up” at the end of the procedure, have been promoted. The duration of pain relief following hypospadias repair was longer with a double caudal injection of bupivacaine than with a single caudal injection by M. Samuel and colleagues (22). Children in the double caudal group received a caudal injection of 0.25% plain bupivacaine, 0.5 ml/kg, and a capped cannula was left *in situ* with an aseptic dressing to deliver a second top-up caudal solution at the completion of the procedures. The second or top-up caudal supplemented and prolonged postoperative analgesia without raising the overall dosage. There was a statistically significant difference in the mean duration of caudal analgesia between 3.45 h for single caudal and 7.85 h for double caudal (*P* < 0.001). Due to the relatively small volumes used in double caudal compared to single or top-up, which may decrease leakage from the caudal epidural space and, therefore, effective prolonged caudal analgesia by 0.25% plain bupivacaine without additives was achieved. However, this would increase the incidence of motor block after double-caudal and risk since the caudal cannula would be inserted into the caudal epidural space.

## 4 Cannulation

Even long-acting local anesthetic drugs such as bupivacaine provide only 4–7 h of analgesia (23–27), caudal catheters may provide both intraoperative stability and better postoperative comfort when the local anesthetic solution is infused or dosed repeatedly. In David Sommerfield's study (28), children who received the caudal catheter experienced postinduction with a bolus of 0.5 ml/kg of 0.25% bupivacaine followed by the postoperative regional regimen of 0.125% bupivacaine with fentanyl 2 ug/ml run at 0.1–0.3 ml kg/h titrated to effect for up to 4 days. Group ‘caudal’ had a single-shot caudal with 1 ml/kg of 0.25% bupivacaine (clonidine) postinduction. They compared the analgesic effectiveness and found that the caudal catheter group had clinically and statistically significant lower interventions for bladder spasm and wound pain than the single caudal in the first 5 days after pediatric ureteric reimplant surgery. Over 75% of caudal catheter patients did not require any bladder spasm intervention on any day. However, this article did not mention the specific time for the first remedial analgesia, which increased the difficulty in evaluating the analgesic prolongation.

More recently, Xu (29) compared the effectiveness between patient-controlled caudal epidural analgesia (PCCA) and patient-controlled intravenous analgesia (PCIA) after peri-anal surgery in adults. Ropivacaine (2 mg/ml) was continuously infused into PCCA group patients at a rate of 4 ml/h. Patients could self-administer 4 ml boluses with a 60-minute lockout time. The medication administered in the PCIA group was sufentanyl and dexmedetomidine. After surgery, patients in the PCCA group had lower VAS scores, less remedial analgesics, and better analgesia satisfaction than those in the PCIA group. Since the medications in the two groups are different, the purpose of this article is to explore an analgesic regimen that can produce sufficient analgesic effects while minimizing opioid intake rather than giving proof that PCCA delivery methods are better than intravenous routes. On the other hand, PCIA is always associated with a relatively high incidence of opioid side effects, including pruritus, nausea, and vomiting.

Similarly, Okonkwo found patients who received single caudal block and intravenous morphine infusions experienced higher pain scores than those with caudal epidural infusions, especially on day 2 (15 vs. 6.5) after delayed primary closure with anterior pelvic osteotomies for the infants aged 5.8 months (range 1.6–17.1 months). This may suggest the superiority of continuous caudal-epidural infusions over additional intravenous morphine. Morphine sparing effect also facilitates children to experience lower rates of gastrointestinal and other related complications (30). Future research is required to investigate whether PCCA could be used for children in a broader sense.

Technically, the constant caudal block is also practical and easy to perform. Sherif M Soaida (31) found it even possible to place an epidural catheter to the thoracic level by caudal approach with ease in children over 10 years old merely based on external landmarks. Nowadays, a purpose-designed set of equipment called the Caudal Extradural Catheter Tray, Oxford Set (B Braun Medical Ltd, Sheffield, UK) has been developed employing a catheter via cannula approach (32). The cannula is mounted on a needle with a 30° beveled tip which facilitates the feeling of passing through the sacrococcygeal ligament, identification of the caudal epidural space, and may reduce the risk of venous cannulation. The three lateral eyes on the extradural catheter maximize the spread of local anesthetic solution and reduce the risk of blockage.

Despite its practicality and efficacy, caudal catheterization suffers from several significant drawbacks. First, caudal catheterization may raise the risk of contamination. In a study (33) of 210 children aged one day to 21 years undergoing genitourinary, orthopedic, and general surgical procedures with combined general and epidural analgesia, 35% (73 of 210) catheters were colonized despite the aseptic technique. Both lumbar (23%; 9 of 40) and caudal (25%; 43 of 170) catheters had similar levels of gram-positive colonization. 16% of the caudal catheters (27 of 170) and 3% of the lumbar catheters (1 of 40) yielded gram-negative organisms that could be cultivated. Fortunately, severe side effects, including meningitis, epidural abscess, or systemic sepsis, are rare. Future studies should explore the appropriate time to remove the catheter before the danger of colonization and systemic infection becomes too high. Second, epidural catheters may limit the children's postoperative activity. Continuous anesthetic infusion may make lower limb numbness and motor weakness more common, which may cause parents’ dissatisfaction (29). Third, the catheter has a high potential for migration, particularly in small infants (34). Although bupivacaine and ropivacaine remain the most commonly used local anesthetic agents for postoperative epidural infusions in infants, clinical concerns exist regarding the potential for local anesthetic systemic toxicity (LAST) with these agents. Continuous chloroprocaine epidural infusion has also been proven an efficacious and safe alternative to the amide local anesthetics on postoperative epidural analgesia for neonates and infants undergoing thoracic, abdominal, and limb procedures (35).

## 5 Adjuvants

### 5.1 S(+)-ketamine

Combinations of ketamine with local anesthetics in children have been shown to prolong the duration of caudal analgesia and decrease the occurrence of ineffective analgesia in several studies (23–27). S(+)-ketamine can be safely performed at 0.5–1.0 mg/kg into the caudal route. However, 0.5 mg/kg is advised by current European standards to minimize its incidence of psychomimetic side effects and concerns regarding neurotoxicity (4, 36). However, utilizing ketamine caudally as an adjuvant in children under the age of one year is not recommended since they are susceptible to the danger of drug-induced increases in apoptosis during the neonatal period and infancy (37).

S(+)-ketamine is the S(+)-enantiomer of racemic ketamine. The anesthetic effect of S(+)-ketamine is twice that of the racemic mixture because of a three-fold greater affinity for the N-methyl-D-aspartate (NMDA) receptor. It doesn't seem that these variations in NMDA receptor affinities correspond to variations in clinical efficacy and dosage necessary. When combined with local anesthetics, racemic ketamine 0.25 mg/ml prolongs analgesia similarly to S(+)-ketamine 0.5 mg/ml (38). The S(+) isomer has fewer side effects, such as less salivation, less cardiac stimulation, less spontaneous motor activity, more excellent analgesia, quicker recovery, fewer psychotomimetic side effects, and a decreased incidence of delirium. Wang (39) also found S(+)-ketamine speeds up orientation recovery and recovery from painless gastroscopy compared to racemic ketamine in Chinese patients.

Several studies have shown S(+)-ketamine used as a caudal additive is highly effective in enhancing the quality and duration of analgesia in children. B. G. Locatelli and his colleagues (38) found that adding 0.5 mg/kg of S(+)-ketamine to caudal levobupivacaine 0.175% significantly prolonged postoperative analgesic duration(167.5 min) in children undergoing abdominal and urological surgery in comparison to caudal levobupivacaine 0.2% and S(+)- ketamine with 0.15% levobupivacaine(94.5 min and 145.5 min). Furthermore, there was no significant difference between the remedial analgesia rate of the last two groups, which indicates that they produced an equal quality of postoperative analgesia, and adding S(+)-ketamine allows a lower concentration of local anesthetic.

NMDA receptors are present throughout the central nervous system, including the lumbar spinal cord, and play an essential role in nociceptive processing. Ketamine is an antagonist at N- methyl-D-aspartate (NMDA) receptors. The analgesic target sites of ketamine may be in the spinal cord. In a randomized, double-blind study, S. J. Martindale and colleagues (40) found that the median analgesic duration was significantly longer with administration of bupivacaine 0.25% (1 ml/kg) combining S(+)-ketamine 0.5 mg/kg caudally(10 h) compared with caudal bupivacaine alone (4.75 h) or intravenous injection of equal S(+)-ketamine plus caudal bupivacaine (4.63 h). Caudal S(+)-ketamine provides more effective analgesia than i.v. S(+)-ketamine, which shows that the main analgesic impact of caudal S(+)-ketamine is a local neuraxial action rather than a systemic one. In another study, Herbert Koinig and colleagues (41) found despite S(+)-ketamine blood levels were significantly lower after caudal administration compared with intramuscular use, caudal S(+)-ketamine provided more effective analgesia, the median[range] analgesia duration of administration S(+)-ketamine caudally and intramuscular was 528 min [220– 1,440 min] and 108 min [62–1,440 min]respectively, indicating a local analgesic effect. Hemodynamic responses were not observed after caudal administration of S(+)-ketamine, which could also be interpreted as having a direct effect on the spinal cord.

There haven't been any reports of significant side effects, such as respiratory depression, cardiovascular alterations, severe neurologic or psychiatric issues, or toxic effects after using S(+)-ketamine. However, an animal study has led to serious worries about how ketamine may affect cortical and spinal neuronal apoptosis in children's developing nervous systems (37, 42).

### 5.2 Opioids

The spinal cord's opioid receptors were discovered, which sparked interest in the epidural delivery of opioids. The administration of epidural opioids has been shown to provide substantiated postoperative analgesia in pediatric practice. The supply of analgesia without the sympathetic or motor block associated with local anesthetics is made possible by injecting opioids into the epidural space. Several studies have provided evidence of the long-lasting profound analgesia produced in children after the administration of caudal epidural morphine. Riddhi Kundu demonstrated the effectiveness of caudal 0.25% bupivacaine with morphine (30–50 mcg/kg) for providing postoperative abdominal analgesia in major laparoscopic surgery in infants and children because of its hydrosoluble potential (43). It has less tachycardia response to port site incision((33 vs. 63% children, *p *= 0.019), produces longer analgesic duration (165 vs. 45 min; *p *= 0.00), and requires less postoperative analgesic than intravenous opioids. Moreover, Magda L Fernandes (44) has provided evidence of synergistic effects in the combination of opioid and local anesthetic caudally. In comparison to the groups receiving caudal bupivacaine with or without clonidine, they discovered that adding morphine 20 μg/kg to 0.166% bupivacaine lowered FLACC pain scores and the amount of analgesics used in the postoperative period of infra-umbilical urological and genital procedures.

Fentanyl crosses the dura and leaves the CSF rapidly because of its high lipid solubility, thereby correlating with its fast onset and short duration of action, making rostral migration less available and fewer incidences of respiratory depression. It has been proved that fentanyl 1 μg/kg with ropivacaine or bupivacaine in the single caudal block can extend the postoperative analgesic duration. A higher dose of 2 μg/kg may cause vomiting and desaturation (45). However, the analgesic efficacy of fentanyl and local anesthetics mixtures in children is still a matter of debate. A study performed by Singh J et al. (46) comparing the effects of fentanyl, clonidine, and ketamine on the duration of caudal analgesia in adjuvant with 0.25% bupivacaine in children revealed that the mean duration of analgesia was even shorter in the fentanyl group (507.75 ± 222.64 min) than plain bupivacaine (529.07 ± 166.00 min). In contrast, Xiong (47) suggested fentanyl is weakly superior to the placebo in this regard.

Despite nausea, vomiting, pruritis, and urinary retention, the main side-effect of epidurally administered opioids is respiratory depression because of medullary respiratory center depression. In Neha Baduni's study (48), respiratory depression was associated with a higher dose of 70 μg/kg morphine caudally in children undergoing lower abdominal and urogenital surgeries, but they all responded to oxygen supplementation, and no naloxone was required. The 30 μg/kg and 50 μg/kg morphine dose was relatively safe for postoperative analgesia.

### 5.3 Other additives

Tramadol is a synthetic weak opioid. In Europe, tramadol is approved and allowed to be used for the therapy of moderate to severe nociceptive pain in children above the age of one to three. In the United States, tramadol is only authorized for use in youth older than 17 (49). According to A Krishnada's study, tramadol extended analgesia by 913 min compared to 438 min in the age group of 1–5 years and scheduled for elective sub-umbilical surgeries (50). Jehan and colleagues show that adding tramadol(1 mg/kg) to a caudal bupivacaine block (0.25%) can attenuate the pro-inflammatory cytokine response, cortisol, and C-reactive protein in children aged 3–7 year undergoing lower abdominal surgery (51). The United States Food and Drug Administration recently released a boxed warning regarding the use of tramadol in children for the risk of respiratory depression (49). Since tramadol has extensive systemic absorption after caudal administration (52), using tramadol as an adjuvant cannot be recommended.

Epinephrine 1:200 000 (5 ug/ml) has been used as a caudal adjuvant for many years. The combination with bupivacaine has been confirmed to extend the duration of pain relief and reduce the incidence of toxic symptoms by delaying absorption from the site of injection and assisting in the early detection of accidental intravenous injections according to its hemodynamic pharmacological effects. However, B Cook (53) demonstrates that its analgesic effect is less pronounced than the addition of ketamine 0.5 mg/kg or clonidine 2 ug/kg to 0.25% bupivacaine on children undergoing inguinal hernia repair and circumcision. Liu's study (54) is the first to examine the effect of local anesthetic with and without epinephrine in caudal anesthesia using a noninvasive continuous cardiac output (CO) monitor in pediatric patients after the administration of a caudal block while under general anesthesia. Epinephrine added to the local anesthetic injected for caudal anesthesia produces significant increases in stroke volume (SV), cardiac index (CI), and CO in children. These hemodynamic changes can occur as early as 7 min after the caudal block. Stroke volume and CI alteration occur only in children 6 months or older when epinephrine is administered to a local anesthetic for caudal anesthesia.

Clonidine probably binds to alpha-2 receptors in the dorsal horn of the spinal cord as adjuvants in caudal blocks. According to a meta-analysis by Wang and Guo, there was no difference between clonidine and the control drug regarding the duration of analgesia for the caudal epidural block for pediatric surgery (55). In Walker et al.'s research, intraspinal injection of local anesthetics and preservative-free clonidine had no significant impact on the spinal cord's apoptotic pattern in newborn rodents (56).

Dexmedetomidine is a highly selective *α*2-adrenergic agonist with an affinity for the *α*2-adrenergic receptor that is eight times that of clonidine (57). In children undergoing inguinal hernia surgery, Q. Xiang showed that caudal bupivacaine combined with dexmedetomidine (1 mg/kg) attenuated the hemodynamic response to hernial sac traction and prolonged the duration of postoperative analgesia in children undergoing inguinal hernia repair(860 min vs. 320 min, *P* < 0.001) (58). Sunil Chiruvella demonstrated that dexmedetomidine provides a longer duration of analgesia than the addition of clonidine, with less requirement of rescue analgesic doses and without any significant differences in the hemodynamic parameters or other side effects (59). Research on animals revealed that dexmedetomidine has neuroprotective benefits through activating imidazoline 1 and 2 receptors and a2 adrenergic receptors (60). The most frequent side effects of using a2 adrenoreceptor agonists are bradycardia and hypotension. However, these symptoms seem less severe in children than in adults.

In terms of the co-administration of bupivacaine and neostigmine caudally, it extended postoperative analgesia but increased the incidence of postoperative nausea and vomiting. According to a meta-analysis, neostigmine extended analgesia by 9.96 h compared to 3.68 h with clonidine and 4.45 h with tramadol (61).

Dexamethasone has also been used as an adjuvant to prolong caudal block duration. A meta-analysis by Matthew showed that single-caudal analgesia lasted 5.43 h longer in patients treated with caudal dexamethasone compared to the control group (62).

The two possible mechanisms of this action are the direct effect on nociceptive fibers and anti-inflammatory properties (63). However, theoretical concerns exist about delayed wound healing, surgical site infection, and hyperglycemia while using it (64). The United States Food and Drug Administration has also issued a warning regarding the epidural injection of corticosteroids. Therefore, the use of corticosteroids as a neuraxial adjuvant in children is not recommended.

The advantages and disadvantages of the various adjuvants are summarized in the table below (Table 1).

**Table 1:** Summary in dose, advantages and disadvantages of caudal additives.

Adjuvant	Dose	Advantages	Disadvantages
S(+)-ketamine	0.5–1 mg/kg	More profound analgesia and fewer side effects compared to racemic ketamine.	Concerns about neurotoxicity in children under 1 year.
Opioids	10–30 *μ*g/kg(morphine) 1 mg/kg(Fentanyl)	Profound analgesia.	Risk of respiratory depression Higher incidence of nausea, vomiting, pruritis, and urinary retention.
Tramadol	–	Weaker pro-inflammatory cytokine response.	The population which could be indicated varies Safety Concern-respiratory depression.
Epinephrine	5 ug/ml	Lower incidence of local anesthetic systemic toxicity Assistance in early detection of accidental intravenous injections.	Analgesic effect less pronounced compared to ketamine(0.5 mg/kg) or clonidine(2 ug/kg) Risk of systemic absorption, cardiovascular effects.
Clonidine	1–2 ug/kg	No significant impact on spinal cord apoptotic pattern in newborn rodents.	Failure to extend analgesic duration of single-caudal significantly Bradycardia and hypotension.
Dexmedetomidine	1–2 ug/kg	Smaller hemodynamic response to hernial sac traction A longer duration of analgesia compared to clonidine Neuroprotective benefits.	Bradycardia and hypotension(Less severe in children compared to adult).
Neostigmine	–	More profound postoperative analgesia than clonidine and tramadol.	Higher incidence of postoperative nausea and vomiting.
Dexamethasone	–	Anti-inflammatory properties.	Delayed wound healing Surgical site infection Hyperglycemia.

Our review has several limitations. Firstly, available studies involved diverse types of infra-inguinal surgery (including abdominal and genitourinary surgery). We are concerned that the degree of postoperative pain is not the same for different types of surgery, which will affect the observation of outcomes. Secondly, we noted differences in the follow-up period and definitions of analgesic duration, which may contribute to heterogeneity. Thirdly, although there are many methods to prolong the analgesic duration of caudal anesthesia, children still have high analgesia scores within 24 h after surgery, which suggests that further exploration is urgently needed.

## 6 Conclusions

Caudal anesthesia is an essential component of perioperative pain management in children of all ages. Extending the duration of caudal analgesia can lessen the untreated acute pain, which may cause patients to fear or even avoid future medical procedures. The information above summarizes current research on the benefits and complications of various approaches in newborns and young children. Multimodal Analgesia approaches such as caudal block combined with patient-controlled analgesia in pediatric patients may provide superior analgesia while minimizing adverse effects, improve patient and family satisfaction, and decrease stress postoperatively. We consider this review to offer anesthesiologists and surgeons additional evidence to evaluate both the advantages and the safety of novel and existing techniques and medicines before routine clinical use to minimize the risk of an unexpected and untoward outcome.
